# Functional Characterization of FeoAB in Iron Acquisition and Pathogenicity in Riemerella anatipestifer

**DOI:** 10.1128/spectrum.01373-23

**Published:** 2023-06-05

**Authors:** Mi Huang, Mengying Wang, Yan Feng, Mingshu Wang, Qun Gao, Dekang Zhu, Renyong Jia, Shun Chen, Xinxin Zhao, Qiao Yang, Ying Wu, Shaqiu Zhang, Bin Tian, Juan Huang, Xumin Ou, Sai Mao, Di Sun, Yu He, Zhen Wu, Anchun Cheng, Mafeng Liu

**Affiliations:** a Engineering Research Center of Southwest Animal Disease Prevention and Control Technology, Ministry of Education of the People’s Republic of China, Chengdu, China; b Key Laboratory of Animal Disease and Human Health of Sichuan Province, Chengdu, China; c International Joint Research Center for Animal Disease Prevention and Control of Sichuan Province, Chengdu, China; d Research Center of Avian Disease, College of Veterinary Medicine, Sichuan Agricultural University, Chengdu, China; Texas A&M University

**Keywords:** *Riemerella anatipestifer*, FeoA, FeoB, ferrous iron transport

## Abstract

The bacterium Riemerella anatipestifer requires iron for growth, but the mechanism of iron uptake is not fully understood. In this study, we disrupted the Feo system and characterized its function in iron import in R. anatipestifer ATCC 11845. Compared to the parent strain, the growth of the Δ*feoA,* Δ*feoB,* and Δ*feoAB* strains was affected under Fe^3+^-limited conditions, since the absence of the *feo* system led to less intracellular iron than in the parent strain. In parallel, the Δ*feoAB* strain was shown to be less sensitive to streptonigrin, an antibiotic that requires free iron to function. The sensitivity of the Δ*feoAB* strain to hydrogen peroxide was also observed to be diminished compared with that of the parent strain, which could be related to the reduced intracellular iron content in the Δ*feoAB* strain. Further research revealed that *feoA* and *feoB* were directly regulated by iron through the Fur regulator and that the transcript levels of *feoA* and *feoB* were significantly increased in medium supplemented with 1 mM MnCl_2_, 400 μM ZnSO_4_, and 200 μM CuCl_2_. Finally, it was shown that the Δ*feoAB* strain of R. anatipestifer ATCC 11845 was significantly impaired in its ability to colonize the blood, liver, and brain of ducklings. Taken together, these results demonstrated that FeoAB supports ferrous iron acquisition in R. anatipestifer and plays an important role in R. anatipestifer colonization.

**IMPORTANCE** In Gram-negative bacteria, the Feo system is an important ferrous iron transport system. R. anatipestifer encodes an Feo system, but its function unknown. As iron uptake may be required for oxidative stress protection and virulence, understanding the contribution of iron transporters to these processes is crucial. This study showed that the Δ*feoAB* strain is debilitated in its ability to import iron and that its intracellular iron content was constitutively low, which enhanced the resistance of the deficient strain to H_2_O_2_. We were surprised to find that, in addition to responding to iron, the Feo system may play an important role in sensing manganese, zinc, and copper stress. The reduced colonization ability of the Δ*feoAB* strain also sheds light on the role of iron transporters in host-pathogen interactions. This study is important for understanding the cross talk between iron and other metal transport pathways, as well as the pathogenic mechanism in R. anatipestifer.

## INTRODUCTION

Iron is necessary for the growth of most bacteria (1), as it is an important cofactor for many proteins involved in respiration, oxidative stress resistance, gene regulation, and other processes (2, 3). Iron exists mainly in two ionic forms, ferric and ferrous, which in turn enables its contribution to various oxidation/reduction processes (4). However, the amount of free iron is very low in vertebrate hosts. In the human body, the majority of iron is bound to heme proteins, such as hemoglobin, iron/sulfur-containing enzymes, iron-binding transport proteins (transferrin), iron-binding glycoproteins (lactoferrin), or the main intracellular iron storage proteins (ferritin) (4–8). Consequently, pathogenic microorganisms have developed multiple high-affinity iron acquisition systems, including siderophore-mediated iron uptake systems, heme uptake systems, and ferrous iron transport systems (1, 9–11). In contrast to insoluble ferric iron, many ferrous iron importers have also been characterized in Gram-negative bacteria, such as Feo systems, MntH, ZupT, EfeUOB, IroT, YfeABCDE, and FutABC (12). Among them, the Feo system is mainly committed to the uptake of ferrous iron and widely exists in prokaryotes (2, 13). Since iron acquisition represents a critical factor in bacterial virulence, mutations in genes involved in iron acquisition or utilization systems may lead to loss of pathogenicity, and these genes could be used as targets for the development of new antibacterial drugs (14–16).

Riemerella anatipestifer is a Gram-negative bacterium belonging to the family *Flavobacteriaceae* that causes serositis and septicemia in domestic ducks and other birds (17). The mortality rate of ducklings infected with the bacterium can reach 75% or even higher (18). At least 21 different serotypes of R. anatipestifer have been identified, with no cross-protection between them (18). Clinical isolates of R. anatipestifer have been found to be resistant to a variety of antibiotics (19, 20). As a result, existing vaccines and antibiotics have limited effectiveness. R. anatipestifer requires iron for growth, and genome sequencing and analysis showed that R. anatipestifer carries several genes related to iron metabolism (21). Our previous study identified the functions of some of these genes. For example, TonB1 and TonB2 are both involved in the utilization of iron/heme, but TonB3 is not (22, 23). The TonB-dependent receptors B739_1208 and B739_1343 were found to be involved in iron utilization and the virulence of R. anatipestifer (24, 25). RhuR (B739_1416) and RhuA (B739_1417) were characterized as a TonB-dependent heme transporter and an outer membrane exposed heme binding protein, respectively (26). The DNA-binding protein from starved cells, Dps, was found to protect R. anatipestifer from oxidative stress damage by chelating excess iron and binding DNA (27). Iron homeostasis in R. anatipestifer is mainly managed by the regulator Fur (28). Sequence analysis showed that R. anatipestifer possesses homologues of the ferrous iron transporters Feo and MntH. In addition to transporting iron, MntH is primarily an importer of Mn^2+^ (12). In this study, we investigated the function of the Feo system in R. anatipestifer. It was shown that the Feo system is essential for Fe^2+^ utilization and is closely linked to metal homeostasis, oxidative stress resistance, and colonization in R. anatipestifer ATCC 11845.

## RESULTS

### Sequence analysis and deletion of *feoA* and *feoB* in R. anatipestifer ATCC 11845.

**FIG 1:**
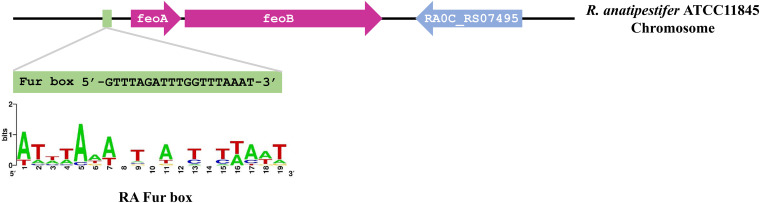
Sequence analysis of FeoA and FeoB in R. anatipestifer ATCC 11845. Schematic of the *feoA* and *feoB* locus in the chromosome of R. anatipestifer ATCC 11845, with gene names and predicted Fur-box (28, 51).

Database searches of the R. anatipestifer ATCC 11845 genome revealed the presence of two predicted ferrous ion transport genes, ferrous iron transport protein A (*feoA*, *RA0C_RS07485*) and ferrous iron transport protein B (*feoB*, *RA0C_RS07490*). The *feoA* and *feoB* genes appear to be in an operonic structure (Fig. 1), and the products of these genes share partial identity at the amino acid level with the Escherichia coli FeoA (43%) and FeoB (54%) proteins, respectively (see Fig. S1 in the supplemental material). To determine the role(s) of the two putative ferrous iron transport proteins, *feoA*, *feoB*, and *feoAB* isogenic mutants were constructed using the natural transformation-based knockout method described in a previous study (29). The deletion of each gene was verified by PCR amplification of each genomic region in the wild-type and deletion strains (Fig. S2A). To confirm that the deletions were nonpolar, total bacterial RNAs were extracted from the wild-type (WT), Δ*feoA*, Δ*feoB*, and Δ*feoAB* strains, and real-time PCR was used to measure the transcript levels of genes downstream of *feoA* and *feoB*. The gene downstream of *feoA* is *feoB*, while the gene downstream of *feoB* is *RA0C_RS07490*, which encodes a GNAT (Gcn5-related *N*-acetyltransferase) family protein. As shown in Fig. S2B, compared to the WT, deletion of *feoA* or *feoB* did not result in transcriptional changes in downstream genes, suggesting that deletion of the *feoA* or *feoB* gene did not cause a polar effect.

### Deletion of *feoA* and *feoB* significantly reduced the growth of R. anatipestifer in the presence of the iron chelator EDDHA.

**FIG 2:**
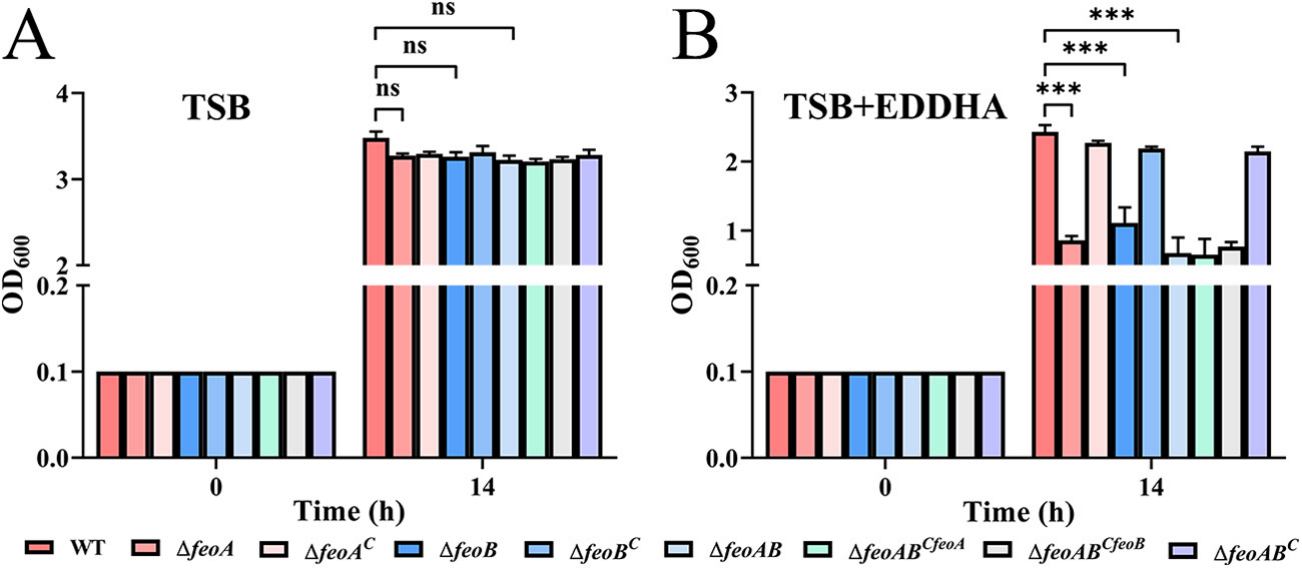
FeoA and FeoB are required for growth under iron-depleted conditions. (A and B) All strains (R. anatipestifer ATCC 11845 pFY02 [WT], Δ*feoA* pFY02 [Δ*feoA*], Δ*feoA* pFY02::*feoA* [Δ*feoA^C^*], Δ*feoB* pFY02 [Δ*feoB*], Δ*feoB* pFY02::*feoB* [Δ*feoB^C^*], Δ*feoAB* pFY02 [Δ*feoAB*], Δ*feoAB* pFY02::*feoA* [Δ*feoAB^CfeoA^*], Δ*feoAB* pFY02::*feoB* [Δ*feoAB^CfeoB^*], Δ*feoAB* pFY02::*feoAB* [Δ*feoAB^C^*]) were grown in 20 mL TSB (A) and in 20 mL TSB containing 40 μM EDDHA (B) at 37°C with shaking starting at an OD_600_ of 0.1. The OD_600_ values were measured at 2-h intervals for 14 h, and representative time points (0 h and 14 h) are shown. All values are the mean (±SD) of three independent experiments. The data are the mean of three independent experiments. Statistical significance was determined by two-way ANOVA (***, *P < *0.001).

To determine whether *feoA* and *feoB* are involved in ferrous iron uptake in R. anatipestifer, we compared the growth ability of R. anatipestifer ATCC 11845 and the Δ*feoA*, Δ*feoB*, and Δ*feoAB* strains in Trypticase soy broth (TSB) and TSB containing the iron chelator ethylenediamine-*N*,*N*′-bis([2-hydroxyphenyl] acetic acid) (EDDHA) at a final concentration of 40 μM. As shown in Fig. 2, there was no significant difference in the growth of the parent strain, three *feo* mutants, and the complemented strains under iron-replete conditions. In contrast, the growth of the Δ*feoA*, Δ*feoB*, and Δ*feoAB* strains was slower than that of the parent strain in TSB supplemented with 40 μM EDDHA (Fig. 2), and growth was restored in the complemented strains (Fig. 2). Notably, the observed growth defect of the Δ*feoAB* strain under iron-limited conditions was only fully restored by introducing pFY02::*feoAB*, but pFY02::*feoA* and pFY02::*feoB* did not revert growth to the wild-type level when introduced into the Δ*feoAB* strain (Fig. 2C). The above-described results showed that all the *feo* mutants exhibited impaired growth in the presence of the iron chelator EDDHA, suggesting that the FeoAB transport system of R. Anatipestifer ATCC 11845 may participate in ferrous iron uptake.

### Deletion of *feoAB* leads to reduced intracellular iron content.

**FIG 3:**
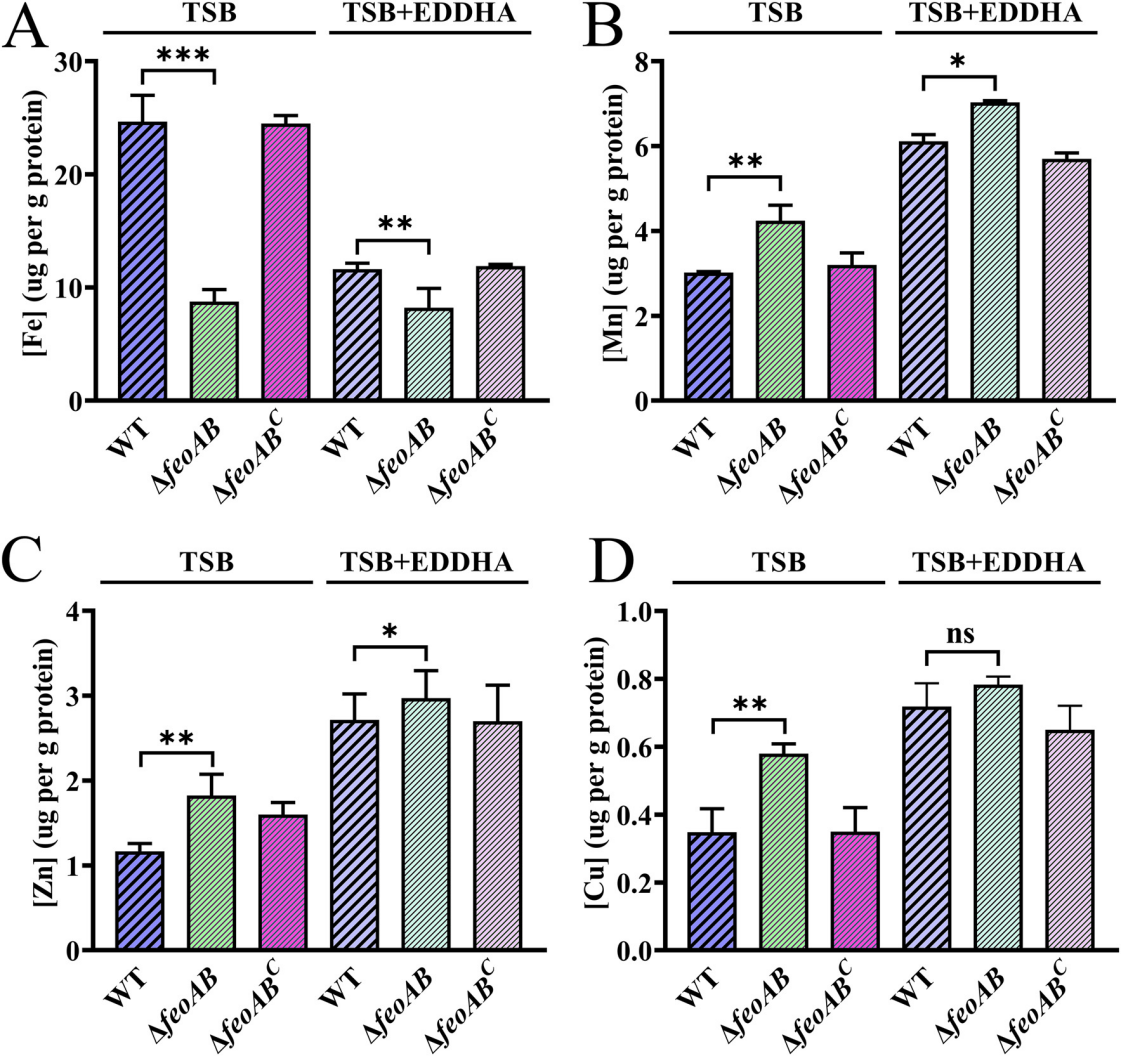
Intracellular metal content (μg/g of protein) of the R. anatipestifer ATCC 11845 and Δ*feoAB* strain cells. (A to D) R. anatipestifer ATCC 11845 pFY02 (WT), R. anatipestifer ATCC 11845 Δ*feoAB* pFY02 (Δ*feoAB*), and R. anatipestifer ATCC 11845 Δ*feoAB* pFY02::*feoAB* (Δ*feoAB^C^*) were grown in 10 mL of TSB and TSB containing EDDHA at 37°C with shaking until the exponential phase, and the intracellular Fe content (A), the intracellular Mn content (B), the intracellular Zn content (C), and the intracellular Cu content (D) were measured by inductively coupled plasma-mass spectrometry. The total concentrations of ions are expressed as μg of ion per gram of protein content, and the values are the mean (±standard deviation) of at least three independent assays. Asterisks indicate statistically significant differences (*, *P < *0.05; **, *P < *0.01; ***, *P < *0.001; ns, not significant).

To characterize the ability of the Feo system to transport iron, the intracellular iron content was determined. R. anatipestifer ATCC 11845 pFY02 (WT), Δ*feoAB* pFY02 (Δ*feoAB*), and Δ*feoAB* pFY02::*feoAB* (Δ*feoAB^C^*) were cultured in TSB medium and TSB supplemented with 40 μM EDDHA, and the intracellular iron content was measured by inductively coupled plasma mass spectrometry (ICP-MS) as described in Materials and Methods. As shown in Fig. 3A, the Δ*feoAB* strain had a significantly lower amount of intracellular iron than the parent strain in both TSB medium and TSB medium supplemented with EDDHA, suggesting that iron uptake is markedly attenuated in a strain lacking *feoAB*. Intracellular levels of other metals were also measured in both TSB medium and TSB medium supplemented with EDDHA. As shown in Fig. 3, the levels of Mn, Zn, and Cu were increased in the Δ*feoAB* strain compared with the parent strain. In addition, the intracellular iron content of all strains under iron-limited conditions decreased, while the levels of other metals (Mn, Zn, and Cu) increased (Fig. 3). The results suggested that the Δ*feoAB* mutation resulted in a decrease in intracellular Fe content and may have contributed to the homeostasis of other metals.

### The *feoAB* mutant strain displayed reduced susceptibility to streptonigrin.

**FIG 4:**
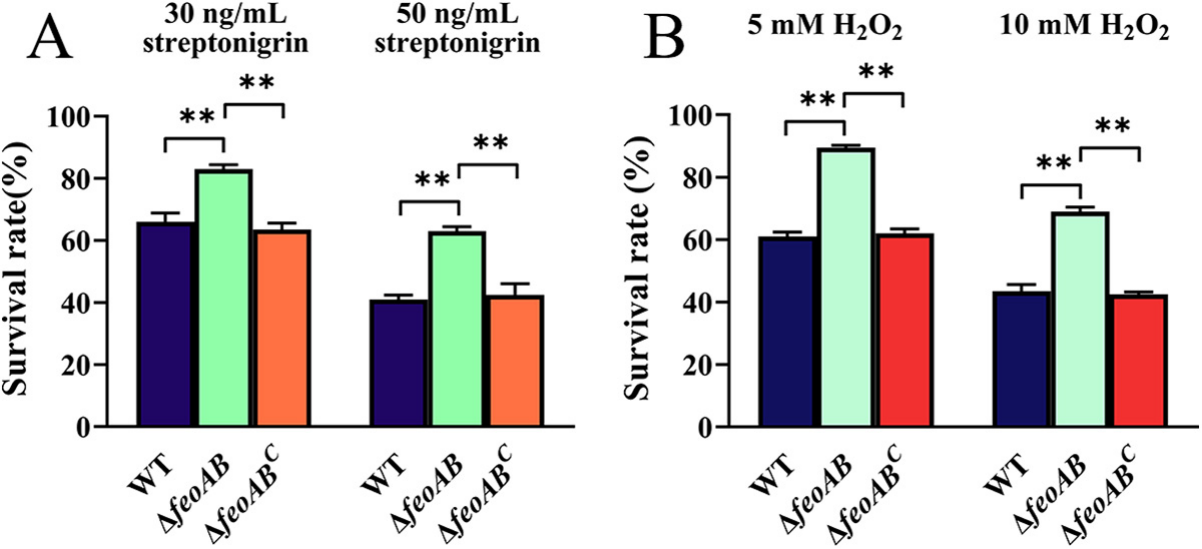
Sensitivity of R. anatipestifer ATCC 11845 and the Δ*feoAB* strain to streptonigrin and H_2_O_2_. R. anatipestifer ATCC 11845 pFY02 (WT), R. anatipestifer ATCC 11845 Δ*feoAB* pFY02 (Δ*feoAB*), and R. anatipestifer ATCC 11845 Δ*feoAB* pFY02::*feoAB* (Δ*feoAB^C^*) were grown in TSB medium at 37°C and 180 rpm until the exponential phase. (A and B) The bacteria were harvested and suspended in PBS at 0.5 OD/mL, and the cell suspensions were exposed to 30 ng/mL streptonigrin and 50 ng/mL streptonigrin (A) and incubated with 5 mM H_2_O_2_ and 10 mM H_2_O_2_ (B) at 37°C for 30 min. The survival rate of each culture was determined as described in Materials and Methods. Error bars represent the standard deviations of three independent experiments and three replicate samples for each experiment. Statistical significance was determined by two-way ANOVA (**, *P < *0.01).

Streptonigrin is a metal-dependent antibiotic that requires intracellular free iron for its bactericidal action through the formation of reactive oxygen radicals (30, 31). Since the intracellular Fe content of the Δ*feoAB* strain was lower than that of the parent strain, we next measured the sensitivity of the Δ*feoAB* strain to streptonigrin. As shown in Fig. 4A, the survival rate of the Δ*feoAB* strain was 2-fold higher than that of the WT after treatment with 30 ng/mL streptonigrin, and the *feoAB* complemented strain showed a recovery of survival rate to the same level as the parental strain. The same change trend was observed after treatment with 50 ng/mL streptonigrin (Fig. 4A). These results suggest that the Δ*feoAB* strain was less susceptible to streptonigrin than the parent strain, which may be due to impaired Feo-dependent ferrous iron transport resulting in reduced intracellular iron levels.

### Deletion of *feoAB* increased the resistance of cells to H_2_O_2_ treatment in comparison with the parent strain.

A major cause of iron toxicity is the cross-reaction of excess intracellular iron with H_2_O_2_ through the Fenton reaction, which results in harmful levels of oxidative stress that affect the growth and viability of bacteria (32). The Δ*feoAB* strain exhibited reduced intracellular iron content, which may lead to an altered response of cells to oxidative stress. Therefore, R. anatipestifer ATCC 11845 pFY02 (WT), Δ*feoAB* pFY02 (Δ*feoAB*), and Δ*feoAB* pFY02::*feoAB* (Δ*feoAB^C^*) were incubated in the presence of 5 mM and 10 mM H_2_O_2_ for 30 min, and the survival rates were recorded as described in Materials and Methods. The WT and Δ*feoAB^C^* exhibited similar survival rates of approximately 60.3% and 61% after exposure to 5 mM H_2_O_2_, respectively (Fig. 4B). However, the survival rate of the Δ*feoAB* strain was higher than that of the parent strain, reaching 88.5% (Fig. 4B). After treatment with 10 mM H_2_O_2_, the survival rate of the Δ*feoAB* strain was still higher than that of the parent strain and the complementation strain (Fig. 4B). These results indicated that knockout of *feoAB* decreased the sensitivity of R. anatipestifer to H_2_O_2_, which may also be related to the decrease in intracellular iron content in the Δ*feoAB* strain.

### The transcription of *feoA* and *feoB* was regulated by iron through Fur and was affected by metal stress.

**FIG 5:**
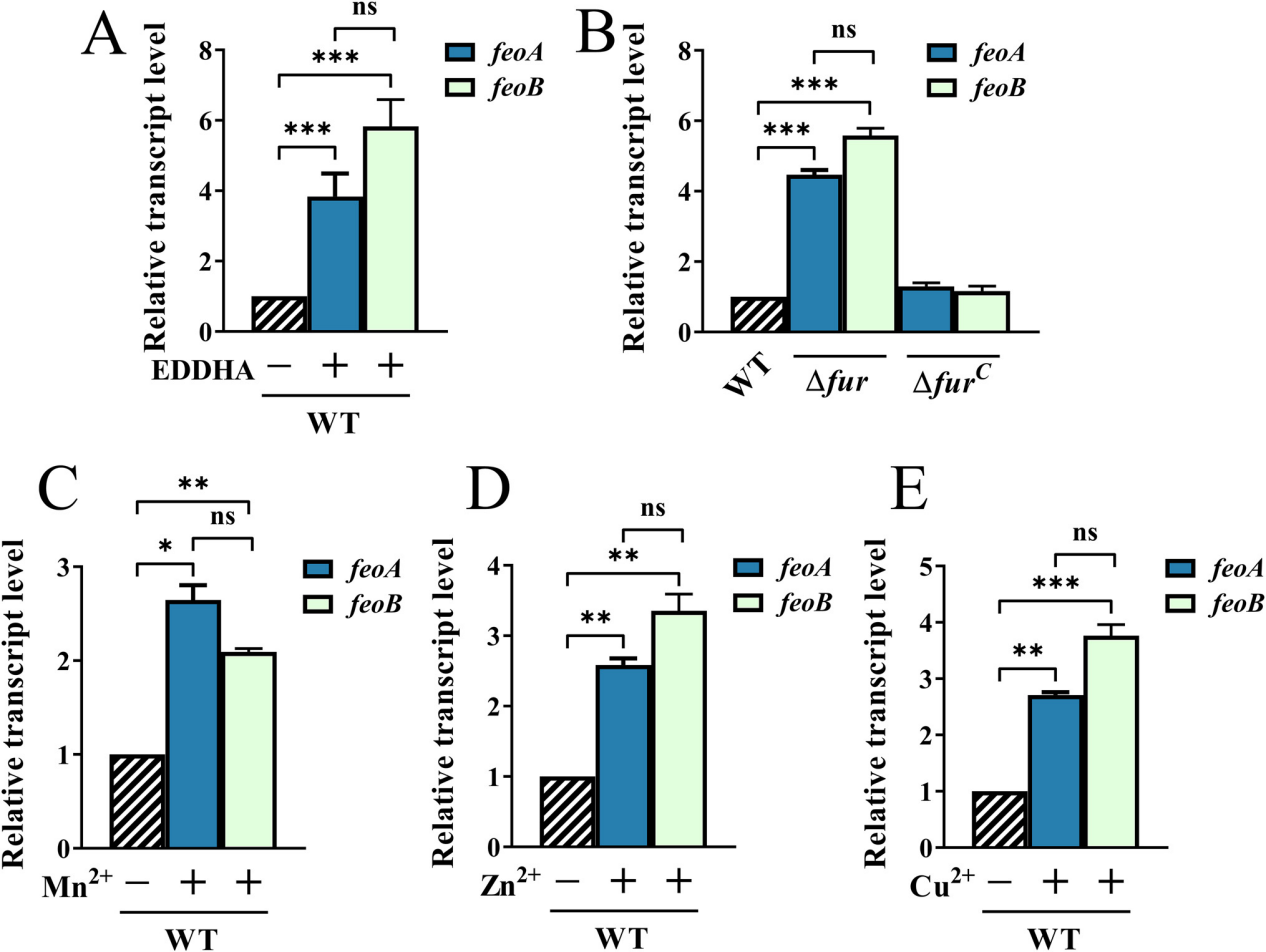
Relative mRNA levels of *feoA* and *feoB* under different conditions. (A) Relative transcript levels of *feoA* and *feoB* in R. anatipestifer ATCC 11845 (WT) grown in TSB and TSB containing 40 μM EDDHA. (B) Relative transcript levels of *feoA* and *feoB* in R. anatipestifer ATCC 11845 (WT), Δ*fur*, and the complementation strain Δ*fur^C^* grown in TSB. (C to E) Relative transcript levels of *feoA* and *feoB* in R. anatipestifer ATCC 11845 (WT) grown in TSB and TSB containing 1 mM MnCl_2_ (C), 400 μM ZnSO_4_ (D), and 200 μM CuCl_2_ (E). The mean values and standard deviations of three independent experiments are shown. Statistical significance was determined by two-way ANOVA (*, *P < *0.05; **, *P < *0.01; ***, *P < *0.001; ns, not significant).

Given that the Feo system is involved in ferrous iron uptake, we next investigated whether the transcription of *feoA* and *feoB* is affected by iron availability. First, we measured the transcript levels of *feoA* and *feoB* in iron-replete medium and iron-depleted medium. The results showed that the transcription levels of *feoA* and *feoB* were ~5-fold higher in cells grown in medium containing 40 μM EDDHA than in cells grown in TSB (Fig. 5A), suggesting that *feoA* and *feoB* could be maximally expressed under iron-limited conditions. In most Gram-negative bacteria, including R. anatipestifer, iron acquisition is regulated by the transcription factor Fur (28). Therefore, we tested the transcription levels of *feoA* and *feoB* in the Δ*fur* strain and its complementation strain. As shown in Fig. 5B, the transcription of *feoA* and *feoB* increased approximately 4- to 5-fold in the Δ*fur* strain compared with the parent strain. Since iron homeostasis can also be affected by other metal stresses (33), we next measured the transcript levels of *feoA* and *feoB* under different metal stresses. The results showed that in response to 1 mM MnCl_2_, 400 μM ZnSO_4_, and 200 μM CuCl_2_, the transcript levels of *feoA* and *feoB* were also significantly increased in R. anatipestifer ATCC 11845 compared to the untreated control (Fig. 5C to E). Taken together, these results indicated that the iron-dependent regulation of *feoA* and *feoB* transcription was mediated by Fur and could be affected by other metals.

### The Feo system contributes to colonization by R. anatipestifer ATCC 11845 in ducklings.

**FIG 6:**
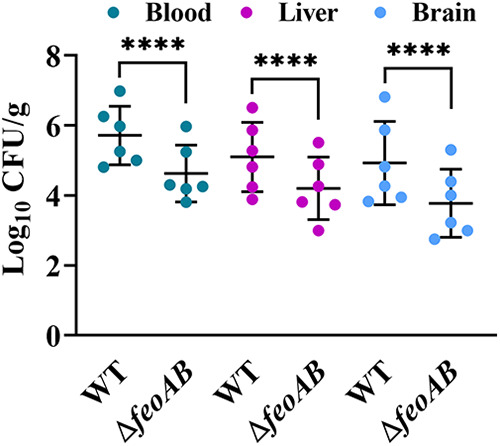
Colonization by R. anatipestifer ATCC 11845 and the Δ*feoAB* strain in the blood, brain, and liver of 3-day-old ducklings. Doses of 10^9^ CFU of R. anatipestifer ATCC 11845 (WT) and the Δ*feoAB* strain were prepared and injected into the leg muscles of 3-day-old ducklings. At 24 h postinfection, bacteria were isolated from the liver, the brain, and the blood of the heart. The data points represent the CFU/g values of the indicated organs in individual animals; the bars show the mean values (*n* = 6). Statistical significance was determined by two-way ANOVA (****, *P < *0.0001).

To investigate whether the Feo system plays a role in the colonization ability of R. anatipestifer ATCC 11845, ducklings were infected with 10^9^ CFU of the parent strain and the Δ*feoAB* strain. The results of bacterial colonization in the blood of the heart, liver, and brain are shown in Fig. 6. While the parent strain colonized at an average of approximately 5 × 10^5^ CFU per gram of the blood of the heart, the Δ*feoAB* strain exhibited a severe defect in its ability to colonize. Similarly, the number of colonies recovered from liver tissues and brain tissues was significantly reduced for the Δ*feoAB* strain compared to the parent strain (*P < *0.0001). The above-described results indicated that the Feo system contributed to colonization by R. anatipestifer ATCC 11845 in ducklings.

## DISCUSSION

Iron is an essential nutrient for most bacterial pathogens, as it serves as a cofactor for numerous enzymes (1). Due to the limited availability of iron in the environment and the host, pathogenic bacteria have evolved numerous mechanisms to acquire iron through iron transport systems, most of which are virulence-associated genes (34). Hence, iron transport systems could potentially promote bacterial survival during infection by facilitating iron transport. In our previous studies, we identified the mechanism of iron transport and the regulation of iron homeostasis, iron efflux, and iron chelation in R. anatipestifer (22, 24–28). To refine the understanding of the mechanism of iron metabolism in R. anatipestifer, the ferrous iron transport system Feo was studied in detail in this study.

The canonical *feo* operon was originally identified in Escherichia coli, and it encodes three proteins, the cytosolic proteins FeoA and FeoC and the integral membrane protein FeoB, which constitute the Feo system (35). FeoA and FeoB are strongly conserved, whereas FeoC is found in only ~13% of operons (36–38). FeoA is a small (~8 kDa) cytoplasmic β-barrel protein, and it has been hypothesized to interact with FeoB to affect functions (2, 36, 39, 40). FeoC is also a small cytoplasmic protein, but it is not widespread among bacteria (36). Although several potential roles for FeoC have been proposed, these roles have not been experimentally tested in more than one species (39). FeoB is a transmembrane protein consisting of a G-protein domain, a GDP dissociation inhibitor (GDI) domain, and a C-terminal transmembrane (TM) domain (41–43). The G-protein domain of FeoB is not only responsible for binding and hydrolyzing GTP or ATP but is also important for Fe^2+^ transport (41, 44, 45). The GDI domain is the link between the G-protein domain and the TM region, which often adopts a novel fold called a “valve” (46). The TM region may serve as a channel region for ferrous iron transport (47, 48).

In the genome of R. anatipestifer ATCC 11845, there were two putative proteins, FeoA and FeoB, which were annotated as ferrous iron transport proteins, but no sequences containing the FeoC domain or a C-rich peptide were found downstream of *feoA* and *feoB*. Mutant strains completely lacking *feoA*, *feoB*, and *feoAB* were generated and analyzed to determine if the absence of *feo* genes affected bacterial growth. The results showed that the loss of *feoA*, *feoB*, and *feoAB* affected growth in defined medium containing restricted iron (in the presence of the iron chelator EDDHA). Intracellular iron content measurement showed a clear distinction between the parent strain and the Δ*feoAB* strain, regardless of the iron-rich condition and the iron-limited condition, indicating that the iron uptake capacity of the Δ*feoAB* strain was weakened. Streptonigrin is an antibiotic that works when the iron concentration inside bacterial cells is high. We found that the Δ*feoAB* strain increased resistance to streptonigrin compared to R. anatipestifer ATCC 11845 (Fig. 4A), which also confirmed the reduction in intracellular iron. Notably, although the Δ*feoAB* strain exhibited lower levels of intracellular iron in TSB medium, the growth of the Δ*feoAB* strain in TSB medium was not affected, which could be an indication that the wild-type strain has an excess of iron beyond what is required for growth.

Although iron is an indispensable nutrient for R. anatipestifer, it is also toxic, as both ferrous and ferric iron are known to induce intracellular ROS formation via Fenton reactions and cause oxidative damage to biomolecules (49). As shown above, the Feo system of R. anatipestifer ATCC 11845 may be a major ferrous iron uptake system and may contribute to elevated iron levels in the bacterial cell, which is related to the oxidative stress response. We compared the survival rates of the Δ*feoAB* strain and the parent strain when exposed to 5 mM and 10 mM H_2_O_2_, and the results showed that the Δ*feoAB* strain had a higher survival rate after incubation with H_2_O_2_ (Fig. 4B), demonstrating that the presence of the Feo system contributes to the oxidative stress sensitivity of R. anatipestifer. This is consistent with the observations made in previous work with Porphyromonas gingivalis (50), showing that the deleterious role of iron contributes to the generation of oxidative stress. Furthermore, iron or manganese could act as a cofactor of antioxidant enzymes such as superoxide dismutase (SodB and SodA). Although the decreased intracellular iron level in the Δ*feoAB* strain may have led to the inactivation of SodB, the increased intracellular manganese level in the Δ*feoAB* strain may have increased *sodA* expression, which could be one of the reasons why the *feoAB*-deficient strain was more resistant to H_2_O_2_.

To determine the regulatory mechanism of the Feo system, we monitored the mRNA levels of *feoA* and *feoB* when the parent strain was grown under different iron conditions to determine whether these genes were regulated by iron. The results showed that the transcript levels of *feoA* and *feoB* were increased under iron-limited conditions, suggesting that the expression of *feoA* and *feoB* was iron-dependent (Fig. 5A). The mechanisms of iron regulation are usually mediated by Fur (28). In this study, we found that *feoA* and *feoB* were also regulated by Fur (Fig. 5B), and the Fur box of R. anatipestifer (51) was present in the promoter regions of *feoAB* (Fig. 1). In the results of intracellular metal content determination, we observed different degrees of increase in intracellular Mn, Zn, and Cu levels (Fig. 3B to D). Although the Δ*feoAB* strain exhibited a decrease in intracellular iron content, we speculated that the cells may also have increased their uptake of other metal ions in unknown ways to help maintain intracellular metal homeostasis. In our previous study, we found that the transcription level of *feo* genes was significantly upregulated in the transcriptome data of R. anatipestifer under excessive manganese conditions (33). Here, the determination of the transcript levels of *feoA* and *feoB* under different metal stresses, such as Mn, Zn, and Cu, revealed that *feoA* and *feoB* were obviously upregulated (Fig. 5C to E). Whether this was a direct effect or was indirectly caused by changes in iron levels is still unknown, and further investigations are warranted to clarify the detailed molecular mechanisms underlying the role of *feoAB* in sensing other metals.

Iron availability is an important determinant of virulence, and genes related to iron metabolism have been extensively studied as candidate virulence factors (24, 25, 27, 28, 52). Indeed, the involvement of the Feo system in bacterial colonization and virulence has also been reported (53–55). Since R. anatipestifer ATCC 11845 is an attenuated strain (56), in this study, we evaluated the effect of deletion of *feoAB* on colonization by R. anatipestifer ATCC 11845 in ducklings. At 1 day after infection, significantly reduced colonization levels were observed in the blood, spleen, and brain of the Δ*feoAB* strain-infected ducklings compared to those of ducklings infected with the wild-type strain (Fig. 6), suggesting that the Δ*feoAB* strain exhibited a severe defect in its ability to colonize.

Given the importance of iron in oxidative stress induction and virulence (55, 57), it is crucial to understand the contribution of the major R. anatipestifer ferrous iron transporter to these processes. Here, we have shown that the *feoA* and *feoB* genes from R. anatipestifer ATCC 11845 encode a functional component of the ferrous iron acquisition system. The Feo system of R. anatipestifer provides the bacteria with an advantage for growth and colonization under the iron-restricted condition of the host by increasing iron uptake and promoting *in vivo* persistence. Overall, this study highlights the importance of ferrous iron acquisition in the pathogenesis of R. anatipestifer and will be useful in identifying novel drug and vaccine targets.

## MATERIALS AND METHODS

### Bacterial strains and plasmids used in this study.

The bacterial strains and plasmids used in this study are listed in Table S1. The primers used in this study are listed in Table S2.

### Media and growth conditions.

E. coli strains were grown in LB at 37°C with shaking at 180 rpm. R. anatipestifer ATCC 11845 strains were grown on LB agar supplemented with 5% sheep blood or in TSB (Solarbio, China) and GC broth (GCB) as described in a previous study (29). EDDHA was purchased from Alfa Chemistry Protheragen, Inc. (USA) and dissolved in distilled water at a concentration of 100 mM. EDDHA was added to the medium to chelate Fe^3+^ (58). Antibiotics were routinely used at the following concentrations (per milliliter): 1 μg erythromycin (Erm) (screening for deletion strains), 1 μg cefoxitin (Cfx) (screening for complementation strains), and 50 μg kanamycin (Kana) (screening for R. anatipestifer strains) (56).

### Construction of the mutant strains.

The mutant strains were constructed based on the natural transformation method as described in a previous study (29). To illustrate this gene deletion method, the *feoA* gene deletion of R. anatipestifer ATCC 11845 was used as an example. The approximately 800-bp upstream sequence and 800-bp downstream sequence of the *feoA* gene were amplified by PCR using the primer pairs *feoA* up P1/*feoA* up P2 and *feoA* down P1/*feoA* down P2, respectively. The 994-bp sequence containing the ErmR cassette was then amplified from the R. anatipestifer CH-1 genome using the primer pair *feoA* erm P1/*feoA* erm P2. The PCR fragments (*feoA* upstream, ErmR cassette and *feoA* downstream) were ligated using the overlap PCR method, and 1 μg of the purified fragments was incubated with 300 μL of R. anatipestifer ATCC 11845 (optical density at 600 nm [OD_600_], 1) for 1 h at 37°C. Mutants were selected on sheep blood plates supplemented with Erm (1 μg/mL) for 2 days at 37°C and identified by PCR. The R. anatipestifer ATCC 11845 Δ*feoB* strain and the R. anatipestifer ATCC 11845 Δ*feoAB* strain were constructed using the same approach.

### Construction of the complementation strains.

The *feoA* gene of R. anatipestifer ATCC 11845 was amplified from the genome of R. anatipestifer ATCC 11845 using the primer pair *feoA* P1/*feoA* P2, containing the XbaI/XhoI restriction sites. The PCR products were purified, digested, and cloned into the shuttle plasmid pFY02 to generate pFY02::*feoA* as described in a previous study (59). The plasmid pFY02::*feoA* was then transformed into Escherichia coli S17-1 cells, and the recombinant plasmid was introduced into the R. anatipestifer ATCC 11845 Δ*feoA* strain by conjugation as described elsewhere (28). Transconjugants were selected on blood agar plates supplemented with Cfx (1 μg/mL) and Kan (50 μg/mL) and identified by PCR amplification.

Similarly, for the construction of pFY02::*feoB* and pFY02::*feoAB*, the primer pairs *feoB* P1/*feoB* P2 and *feoAB* P1/*feoAB* P2 were used. The primers *feoB* P1 and *feoAB* P1 contained the StuI restriction sites, and the primers *feoB* P2 and *feoAB* P2 contained the XhoI restriction sites. The plasmid pFY02::*feoB* was introduced into the R. anatipestifer ATCC 11845 Δ*feoB* strain to produce R. anatipestifer ATCC 11845 Δ*feoB* pFY02::*feoB* (Δ*feoB^C^*). The plasmid pFY02::*feoAB* was introduced into the strain R. anatipestifer ATCC 11845 Δ*feoAB* to generate R. anatipestifer ATCC 11845 Δ*feoAB* pFY02::*feoAB* (Δ*feoAB^C^*). R. anatipestifer ATCC 11845 Δ*feoAB* pFY02::*feoA* (Δ*feoAB^CfeoA^*) and R. anatipestifer ATCC 11845 Δ*feoAB* pFY02::*feoB* (Δ*feoAB^CfeoB^*) were constructed using the same approach.

### *In vitro* growth curve determination.

The *in vitro* growth curves of the tested strains were determined by measuring the OD_600_ using a spectrophotometer (Eppendorf BioPhotometer, Germany). The strains R. anatipestifer ATCC 11845 pFY02 (WT), R. anatipestifer ATCC 11845 Δ*feoA* pFY02 (Δ*feoA*), R. anatipestifer ATCC 11845 Δ*feoB* pFY02 (Δ*feoB*), R. anatipestifer ATCC 11845 Δ*feoAB* pFY02 (Δ*feoAB*), R. anatipestifer ATCC 11845 Δ*feoA* pFY02::*feoA* (Δ*feoA^C^*), R. anatipestifer ATCC 11845 Δ*feoB* pFY02::*feoB* (Δ*feoB^C^*), R. anatipestifer ATCC 11845 Δ*feoAB* pFY02::*feoA* (Δ*feoAB^CfeoA^*), R. anatipestifer ATCC 11845 Δ*feoAB* pFY02::*feoB* (Δ*feoAB^CfeoB^*), and R. anatipestifer ATCC 11845 Δ*feoAB* pFY02::*feoAB* (Δ*feoAB^C^*) were all cultured in 20 mL of TSB medium and TSB medium supplemented with 40 μM EDDHA at an OD_600_ of 0.1, and growth rates were determined at 37°C and 180 rpm by measuring the OD_600_ every 2 h for 14 h. Experiments were performed in triplicate using three biological replicates.

### Measurement of metal content.

ICP-MS analyses were used to measure the metal content in R. anatipestifer strains as described in previous studies (33). Briefly, R. anatipestifer ATCC 11845 pFY02, R. anatipestifer ATCC 11845 Δ*feoAB* pFY02, and R. anatipestifer ATCC 11845 Δ*feoAB* pFY02::*feoAB* were grown in 10 mL of TSB or TSB containing EDDHA at 37°C for 4 to 6 h until the exponential phase. Cells were washed with phosphate-buffered saline (PBS) containing 0.1 M EDTA. Cell pellets were then resuspended in 400 μL of ultrapure water and lysed using a FastPrep-96 automated homogenizer (MP Biomedicals). The cytosolic content was separated by centrifugation, the total protein concentration was measured using a bicinchoninic acid (BCA) protein assay kit (Thermo Scientific, Waltham, MA), and the supernatants were stored at −80°C for 24 h. Then, 600 μL of 5% HNO_3_ with 0.1% Triton X-100 was added to the supernatants. Samples were incubated at 95°C for 30 min and centrifuged at 12,000 rpm for 10 min, and the supernatants were diluted to 2% HNO3 for detection. The metal content was analyzed by ICP-MS (Elan DRC II, Perkin-Elmer) using Ga as an internal standard. The total ion concentration is expressed as micrograms of ion per gram of protein. Triplicate cultures of each strain were analyzed in a single experiment, and the experiment was repeated at least three times.

### Streptonigrin sensitivity assay.

The streptonigrin resistance assay was performed as previously described (28). Briefly, the tested strains (R. anatipestifer ATCC 11845 pFY02, R. anatipestifer ATCC 11845 Δ*feoAB* pFY02, and R. anatipestifer ATCC 11845 Δ*feoAB* pFY02::*feoAB*) were cultured in TSB medium overnight, and then the bacteria were grown in fresh TSB at 37°C and 180 rpm until the exponential phase (OD_600_, approximately 1 to 1.5). Cells were harvested by centrifugation at 6,000 rpm for 10 min, and pellets were diluted with sterile PBS up to an OD_600_ of 0.5 in a volume of 1 mL. Streptonigrin (Sigma-Aldrich, St. Louis, USA) was diluted to 1 μg/mL with sterile PBS and added to each tube of bacterial solution at final concentrations of 0 ng/mL, 30 ng/mL, and 50 ng/mL. After incubation at 37°C for 30 min, the bacterial solution was diluted and plated on GCB plates for colony counting (designated T0, T30, and T50). After incubation at 37°C for 24 h, the grown colonies were counted. The survival rate was calculated as (T30/T0) × 100% and (T50/T0) × 100%. Experiments were performed in triplicate using three biological replicates.

### H_2_O_2_ challenge.

For H_2_O_2_ challenge, bacterial cells were prepared as described in a previous study (27, 28). Briefly, the strains (R. anatipestifer ATCC 11845 pFY02, R. anatipestifer ATCC 11845 Δ*feoAB* pFY02, and R. anatipestifer ATCC 11845 Δ*feoAB* pFY02::*feoAB*) were grown in TSB medium to the exponential phase and washed twice in PBS. The cell suspension was diluted to an OD_600_ of 0.5 in a volume of 1 mL, and 5 mM H_2_O_2_ and 10 mM H_2_O_2_ were added to the samples. The same volume of PBS was added to the control group. The suspension was incubated at 37°C for 30 min. After incubation, the above-described samples were diluted and plated on GCB plates for CFU counting. Colonies from the control and experimental groups were counted, and the number of living bacteria was expressed as T0, T5, and T10. Survival rates were expressed by (T5/T0) × 100% and (T10/T0) × 100%. Experiments were performed in triplicate using three biological replicates.

### Real-time PCR.

For RNA extraction, the strain R. anatipestifer ATCC 11845 was grown in TSB medium and TSB medium containing 40 μM EDDHA, 1 mM MnCl_2_, 400 μM ZnSO_4_, and 200 μM CuCl_2_. R. anatipestifer ATCC 11845 and R. anatipestifer ATCC 11845 Δ*fur* were grown in TSB medium. All the strains were grown at 37°C and 180 rpm until the exponential phase. Then, 6 × 10^9^ CFU (1 OD_600_ = 2 × 10^9^ CFU) (22) of the cells was mixed with 1 mL RNAprotect bacterial reagent (Qiagen, 76506), and the total RNA was isolated using the RNeasy Protect Bacteria minikit (Qiagen, 74524) as described in a previous study (28). cDNA was synthesized from 1,000 ng template RNA with HiScript QRT SuperMix for quantitative PCR (qPCR) (Vazyme, R123-01). The cDNA was quantified by real-time PCR using SYBR green master mix (Vazyme, Q111-01) on a CFX Connect real-time system (Bio-Rad), and the mRNA levels of the *feoA* and *feoB* genes were normalized to those of 16S rRNA as described in a previous study (60). Experiments were performed in triplicate using three biological replicates.

### Colonization assays.

For bacterial colonization assays, R. anatipestifer ATCC 11845 and the R. anatipestifer ATCC 11845 Δ*feoAB* strain were grown in 50 mL of TSB (with the initial OD adjusted to 0.1) at 37°C with shaking until the exponential phase. The bacteria were then collected by centrifugation at 6,000 rpm for 10 min, resuspended in 10 mL of PBS, and centrifuged again. This process was repeated three times to wash the bacteria. The OD_600_ values of the bacterial suspensions were measured, and the suspensions were adjusted to 5 × 10^9^ CFU/mL (1 OD_600_ = 2 × 10^9^ CFU) (22). Ducklings (3 days old) were infected intramuscularly with R. anatipestifer ATCC 11845 and the R. anatipestifer ATCC 11845 Δ*feoAB* strain (10^9^ CFU/duckling, 10 ducklings/group). At 24 h postinfection, six ducklings in each group were randomly selected and euthanized by forced CO_2_ inhalation. Liver, brain, and blood from the heart were collected in sterile Whirl-Pak bags (Nasco, B01245WA, USA), weighed, and added to PBS (0.1 g of sample added to 0.9 mL of PBS). All the samples were then transferred to 100 sterile tubes, homogenized using a FastPrep-24 instrument (MP, USA), serially diluted in PBS, and plated on blood agar plates supplemented with 50 μg/mL Kana (for R. anatipestifer ATCC 11845) or on blood agar plates supplemented with 50 μg/mL Kana and 1 μg/mL Erm (for R. anatipestifer ATCC 11845 Δ*feoAB*). The plates were incubated overnight at 37°C to determine the bacterial CFU count.

### Ethics approval.

The animal study was reviewed and approved by the local animal welfare authorities and the ethics committee of Sichuan Agricultural University. The 1-day-old ducklings were purchased from Grimaud Farms in Chengdu, Sichuan, China, and housed in our animal facilities with free access to food and water.

### Statistical analysis.

All experimental data are expressed as the mean ± 1 standard deviation (SD). Statistical analysis was performed using Prism 8 (GraphPad Software, California, USA). Independent Student’s *t* test was used to compare two groups, and one-way analysis of variance (ANOVA) or two-way ANOVA was used to compare multiple groups. A *P* value of <0.05 was considered significant.

### Data availability.

The nucleotide sequences of R. anatipestifer ATCC 11845 are deposited in GenBank under accession number CP003388.1. The GenBank accession numbers of the Feo system and its homologues are as follows: WP_004916263.1 for FeoA of R. anatipestifer ATCC 11845, NP_417867.1 for the homologue in Escherichia coli, WP_014411322.1 for FeoB of R. anatipestifer ATCC 11845, and NP_417868.1 for the homologue in Escherichia coli.
