# Supplementary material for: Functional Characterization of FeoAB in Iron Acquisition and Pathogenicity in Riemerella anatipestifer
Source: Microbiol Spectr. 2023 Jun 5;11(4):e01373-23. doi: 10.1128/spectrum.01373-23 (PMC10434265; doi:10.1128/spectrum.01373-23)
Supplement: Supplemental file 1 — Table S1 to S2. Download spectrum.01373-23-s0001.docx, DOCX file, 0.02 MB [file spectrum.01373-23-s0001.docx]

Supplemental file 1

**Table S1. Strains and plasmids used in this study.**

| ***Riemerella anatipestifer* strains** | **Genotype** | **Source or reference** |
| --- | --- | --- |
| *R. anatipestifer* ATCC11845 | ATCC11845 | Laboratory collection |
| *R. anatipestifer* CH-1 | Serotype 1 | Laboratory collection |
| *R. anatipestifer* ATCC11845 Δ*feoA* | *R. anatipestifer* *feoA*,ErmR | This study |
| *R. anatipestifer* ATCC11845 Δ*feoB* | *R. anatipestifer* *feoB*,ErmR | This study |
| *R. anatipestifer* ATCC11845 Δ*feoAB* | *R. anatipestifer* *feoAB*,ErmR | This study |
| *R. anatipestifer* ATCC11845 Δ*fur* | *R. anatipestifer* *fur*,ErmR | Laboratory collection |
| *R. anatipestifer* ATCC11845 pFY02 | *R. anatipestifer* carrying pFY02*,* ErmR,CfxR | This study |
| *R. anatipestifer* ATCC11845 Δ*feoA* pFY02 | *R. anatipestifer* Δ*feoA* carrying pFY02*,*ErmR,CfxR | This study |
| *R. anatipestifer* ATCC11845 Δ*feoB* pFY02 | *R. anatipestifer* Δ*feoB* carrying pFY02*,*ErmR,CfxR | This study |
| *R. anatipestifer* ATCC11845 Δ*feoAB* pFY02 | *R. anatipestifer* Δ*feoAB* carrying pFY02*,*ErmR,CfxR | This study |
| *R. anatipestifer* ATCC11845 Δ*feoA* pFY02::*feoA* | *R. anatipestifer* Δ*feoA* carrying pFY02::*feoA,*ErmR,CfxR | This study |
| *R. anatipestifer* ATCC11845 Δ*feoB* pFY02::*feoB* | *R. anatipestifer* Δ*feoB* carrying pFY02::*feoB,*ErmR,CfxR | This study |
| *R. anatipestifer* ATCC11845 Δ*feoAB* pFY02::*feoA* | *R. anatipestifer* Δ*feoAB* carrying pFY02::*feoA,*ErmR,CfxR | This study |
| *R. anatipestifer* ATCC11845 Δ*feoAB* pFY02::*feoB* | *R. anatipestifer* Δ*feoAB* carrying pFY02::*feoB,*ErmR,CfxR | This study |
| *R. anatipestifer* ATCC11845 Δ*feoAB* pFY02::*feoAB* | *R. anatipestifer* Δ*feoAB* carrying pFY02::*feoAB,*ErmR,CfxR | This study |
| **Plasmids** | **Genotype** | **Source or reference** |
| pFY02 | *ermF* promoter, *ori*ColE1, *ori* pRA0726*,*  Ap^R^, Cfx^R^ | (Feng et al., 2018) |
| pFY02::*feoA* | Plasmid pFY02 with *feoA* insert | This study |
| pFY02::*feoB* | Plasmid pFY02 with *feoB* insert | This study |
| pFY02::*feoAB* | Plasmid pFY02 with *feoAB* insert | This study |

ErmR, erythromycin resistance; CfxR, cefoxitin resistance.

**Table S2. Primers used in this study**

| **Primer** | **Organism** | **Sequence (5’–3’)** |
| --- | --- | --- |
| *feoA* up P1 | RA ATCC11845 | CGTCTTCTAGCGTTACCGAAGCATCG |
| *feoA* up P2 | RA ATCC11845 | CTTCGTAAGACTGGAAAGTGGTGGCTATCATCATAGCCCAAAACCCTAC |
| *feoA* down P1 | RA ATCC11845 | CATCCTTCGTAGTTCAAAGTCGCGCTCTTAGAGAAGAAGAAGCGG |
| *feoA* down P2 | RA ATCC11845 | GAGGAGTTCTAGATATCACCTCAGC |
| *feoA* erm P1 | RA CH-1 | GGTAGGGTTTTGGGCTATGATGATAGCCACCACTTTCCAGTCTTACGAAG |
| *feoA* erm P2 | RA CH-1 | CCGCTTCTTCTTCTCTAAGAGCGCGACTTTGAACTACGAAGGATG |
| *feoA* P1 | RA ATCC11845 | GCTCTAGAGCATGAAATCCACAGCAGAAG |
| *feoA* P2 | RA ATCC11845 | CCGCTCGAGCGGTTACATTGGTTCCACTAG |
| *feoB* up P1 | RA ATCC11845 | GTAGGTATGGCGAGTAATCTTACATGG |
| *feoB* up P2 | RA ATCC11845 | CTTCGTAAGACTGGAAAGTGGTGGACTTCCCTACATTAGGATTTCC |
| *feoB* down P1 | RA ATCC11845 | CATCCTTCGTAGTTCAAAGTCGCCATAGAACCAGCGATTAGTCC |
| *feoB* down P2 | RA ATCC11845 | GGCAAGCAACGCCTCTAAAGCTGGAC |
| *feoB* erm P1 | RA CH-1 | GGAAATCCTAATGTAGGGAAGTCCACCACTTTCCAGTCTTACGAAG |
| *feoB* erm P2 | RA CH-1 | GGACTAATCGCTGGTTCTATGGCGACTTTGAACTACGAAGGATG |
| *feoB* P1 | RA ATCC11845 | AAGGCCTTATGATGAAGGAAGATAAGACCAC |
| *feoB* P2 | RA ATCC11845 | CCGCTCGAGCGGTTACTTGAATATTTGATACAC |
| *feoAB* up P1 | RA ATCC11845 | CGTCTTCTAGCGTTACCGAAGCATCG |
| *feoAB* up P2 | RA ATCC11845 | CTTCGTAAGACTGGAAAGTGGTGGCTATCATCATAGCCCAAAACCCTACC |
| *feoAB* down P1 | RA ATCC11845 | CATCCTTCGTAGTTCAAAGTCGCCATAGAACCAGCGATTAGTCC |
| *feoAB* down P2 | RA ATCC11845 | GGCAAGCAACGCCTCTAAAGCTGGAC |
| *feoAB* erm P1 | RA CH-1 | GGTAGGGTTTTGGGCTATGATGATAGCCACCACTTTCCAGTCTTACGAAG |
| *feoAB* erm P2 | RA CH-1 | GGACTAATCGCTGGTTCTATGGCGACTTTGAACTACGAAGGATG |
| *feoAB* P1 | RA ATCC11845 | AAGGCCTTATGAAATCCACAGCAGAAG |
| *feoAB* P2 | RA ATCC11845 | CCGCTCGAGCGGTTACTTGAATATTTGATACAC |
| 16SrRNA P1 | RA ATCC11845 | ATGCGAAAGGAGGATTGC |
| 16SrRNA P2 | RA ATCC11845 | TTACACCTCAAATACCTC |
| *recA* RT-PCR P1 | RA ATCC11845 | TGAAACTAGGTGATGGTACG |
| *recA* RT-PCR P2 | RA ATCC11845 | GGGTAGGTGGTTATCCTAAG |
| *feoA* RT-PCR P1 | RA ATCC11845 | ATTATGGAAATGGGCTTGCT |
| *feoA* RT-PCR P2 | RA ATCC11845 | GGTTCCACTAGAATATAAGCCG |
| *feoB* RT-PCR P1 | RA ATCC11845 | ACCGTTTATGACTTGTGCTG |
| *feoB* RT-PCR P2 | RA ATCC11845 | GAACCATCGCTCTATAACTCAC |

**REFERENCE**

Feng, Y., Cheng, A., and Liu, M. (2018). [Construction and application of Escherichia Coli-Riemerella anatipestifer efficient shuttle plasmid pFY02]. *Sheng Wu Gong Cheng Xue Bao* 34**,** 1596-1605.
