# Supplementary material for: Functional Characterization of FeoAB in Iron Acquisition and Pathogenicity in Riemerella anatipestifer
Source: Microbiol Spectr. 2023 Jun 5;11(4):e01373-23. doi: 10.1128/spectrum.01373-23 (PMC10434265; doi:10.1128/spectrum.01373-23)
Supplement: Supplemental file 2 — Fig. S1 and S2. Download spectrum.01373-23-s0002.docx, DOCX file, 0.9 MB [file spectrum.01373-23-s0002.docx]

Supplemental file 2

**Fig S1. Multiple sequence alignment of FeoA and FeoB.**

**
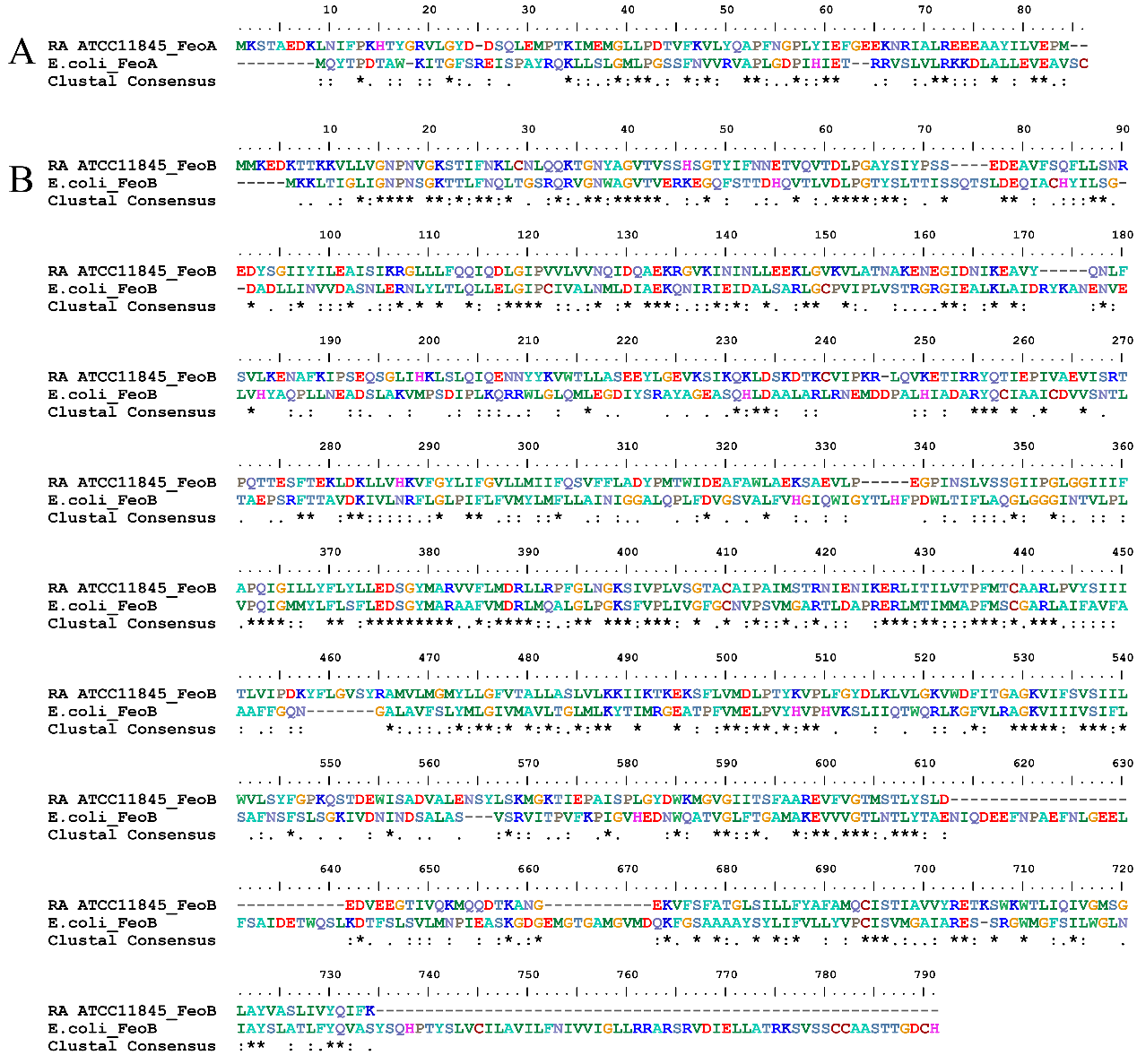
**

**Fig S1. Multiple sequence alignment of FeoA and FeoB.** Multisequence alignment of FeoA and FeoB from *R. anatipestifer* ATCC11845 and *E. coli*. Protein sequences were aligned using MEGA software. Identical residues are present as marked with an asterisk (*).

**Fig S2. Construction of *feo* deletion strains.**

**
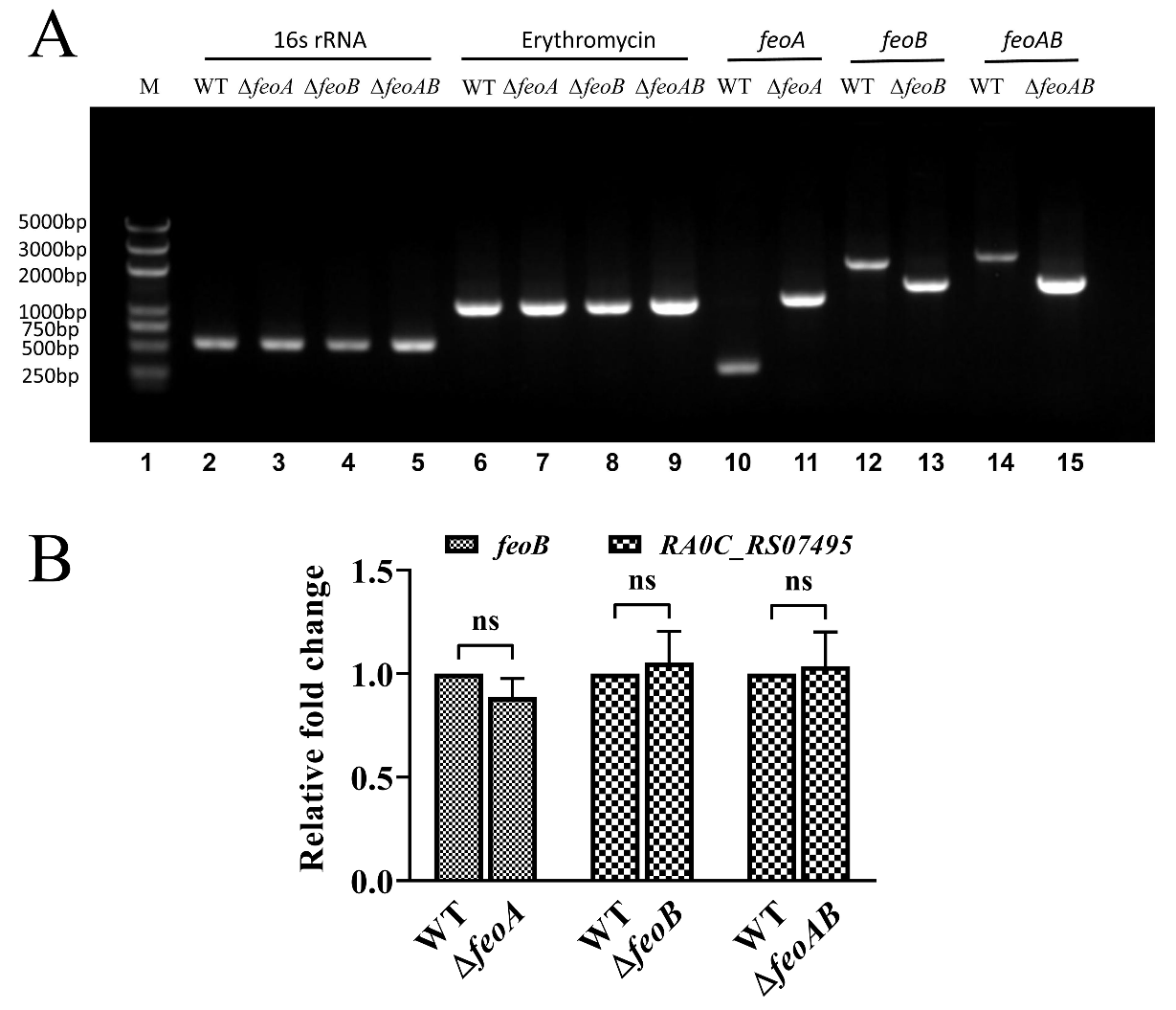
**

**Fig S2. Construction of *feo* deletion strains. (A)** Deletion of the *feo* genes was verified by PCR and agarose gel electrophoresis. The 16S rRNA sequence (~500 bp) and the erythromycin sequence (~1000 bp) were amplified from the mutant strains (16S rRNA, lane 3-lane 5; erythromycin, line 7-line 9) and RA ATCC11845 (lane 2 and lane 6). In the deletion strains, *feo* genes were replaced by the resistance gene, with an ~1100-bp product of the Δ*feoA* strain (line 11), an ~1400-bp product of the Δ*feoB* strain (line 13) and an ~1450-bp product of the Δ*feoAB* strain (line 15). M indicates the DNA marker. **(B)** No polar effect from deletion of *feoA*, *feoB* and *feoAB*. Transcript levels of genes downstream of *feoA* and *feoB* were determined by qRT‒PCR in the WT, Δ*feoA*, Δ*feoB* and Δ*feoAB* strains. Mean values and standard deviations of three independent experiments are shown, ns means not significant.
